# Clinical Characteristics of Patients With Anti‐Signal Recognition Particle Antibody: A Cohort Study

**DOI:** 10.1002/iid3.70297

**Published:** 2026-01-11

**Authors:** Ameen Jubber, Maumer Durrani, Abdullah Almayahi, Kehinde Sunmboye

**Affiliations:** ^1^ University Hospitals of Leicester Leicester UK; ^2^ University of Leicester Leicester UK

**Keywords:** anti‐SRP antibody, extramuscular manifestations, immune mediated necrotizing myopathy, inflammatory myopathy, interstitial lung disease, myositis‐specific autoantibodies

## Abstract

**Background:**

Anti‐signal recognition particle (anti‐SRP) antibodies are myositis‐specific autoantibodies associated with immune‐mediated necrotizing myopathy. This study was undertaken to better understand how patients with anti‐SRP antibodies have been managed at our tertiary centre and to assess the spectrum of clinical features and outcomes in routine clinical practice.

**Methods:**

We conducted a retrospective evaluation of 25 patients with anti‐SRP antibodies identified via line‐blot immunoassay at a tertiary care centre (2019–2024). Demographic, clinical, serological, and imaging data were reviewed.

**Results:**

The group of patients had a mean age of 60.1 years, with a female‐to‐male ratio of 10:15. Eight patients (32%) were diagnosed with myositis, primarily presenting with proximal muscle weakness. Interstitial lung disease was observed in 53% of the group of patients, and 50% of the subset of patients with myositis. Coexisting myositis‐specific autoantibodies were present in 32%, and 48% had positive antinuclear antibody titres (≥ 1:400). Cardiac involvement was reported in two myositis patients. Corticosteroids, often combined with mycophenolate mofetil or other immunosuppressants, formed the basis of treatment.

**Conclusion:**

Anti‐SRP antibodies are associated with a heterogeneous clinical spectrum, with many patients lacking myositis features. There was a high prevalence of coexisting autoantibodies. Further studies are needed to elucidate the pathogenic role of anti‐SRP and optimise management strategies.

## 1 Introduction

Anti‐signal recognition particle (anti‐SRP) antibodies are a subset of myositis‐specific autoantibodies (MSAs) primarily associated with immune‐mediated necrotizing myopathy (IMNM) [1]. Described first by Reeves et al. in 1986 in the serum of a patient with polymyositis [2], these autoantibodies target SRP, a cytoplasmic RNA protein involved in regulating the translocation of proteins that are newly synthesised across the endoplasmic reticulum [3].

Given its importance in cellular protein trafficking, the disruption of SRP function by autoantibodies is hypothesised to cause muscle pathology [4]. Unlike other forms of idiopathic inflammatory myopathy (IIM), IMNM associated with anti‐SRP antibodies is characterised histologically by widespread muscle fibre necrosis with minimal or absent inflammatory infiltrates [5].

The pathophysiological mechanisms underlying anti‐SRP myopathy likely involves several processes, including the antibody targeting skeletal muscle fibres, resulting in impaired myoblast regeneration, myofiber atrophy, and increased reactive oxygen species [6]. Other mechanisms such as endoplasmic reticulum stress/autophagy may also play a significant role [4].

Clinically, anti‐SRP‐associated IMNM presents with acute or subacute onset of severe proximal muscle weakness, often accompanied by markedly elevated serum creatine kinase (CK) levels. Furthermore, extramuscular manifestations such as interstitial lung disease (ILD) and cardiac complications have been reported [5].

Detection of anti‐SRP antibodies is essential for the diagnosis and management of IMNM, enabling early recognition and the initiation of immunosuppressive therapy. Anti‐SRP myopathy has been associated with a more severe disease course compared to other forms of myositis, with multiple relapses [7]. However, recent evidence has shown a more diverse clinical spectrum [3]. This shows the need for further evaluation to clarify the significance of anti‐SRP positivity in different clinical contexts.

The 224th European Neuromuscular Centre (ENMC) International Workshop provided a standardised approach to IMNM, identifying anti‐SRP myopathy as one of the most disabling autoimmune myopathies, with an initially severe muscle weakness and poor muscle recovery even following treatment. Of the extramuscular manifestations, the most concerning is the risk of cardiac involvement [7].

In this retrospective service evaluation, we aim to describe the clinical characteristics, associated autoantibodies, and treatment outcomes in a group of patients with anti‐SRP antibodies identified at a tertiary care centre. By comparing our observations with previously published studies, we seek to contribute to the growing body of literature on anti‐SRP‐associated diseases.

## 2 Methods

This retrospective study was conducted at University Hospitals of Leicester, where all patients tested for MSAs between 2019 and 2024 were identified through the electronic results system. In total, 25 patients tested positive for anti‐SRP antibody, and these patients were included in the evaluation. Clinical information was obtained from electronic medical records, including demographic data, clinical features, laboratory results, imaging findings, treatment details, and outcomes.

### 2.1 Antibody Detection Methods

Anti‐SRP antibodies, along with other MSAs, were tested using a commercially available line‐blot immunoassay as part of the extended myositis panel. This method involves immobilised antigenic proteins on a nitrocellulose membrane, where patient serum is incubated, and any bound antibodies are detected using enzyme‐conjugated secondary antibodies. Line‐blot immunoassays are commonly used for detecting MSAs, but the potential for false positives is a recognised limitation of this platform, and inconsistent results for some antigens have been seen among different studies in the literature. Therefore, a discussion on the reliability of line‐blot assays and the possibility of false‐positive results is provided in the discussion section.

In our centre, line‐blot immunoassay results are reported as: positive, negative or equivocal only; semi‐quantitative signal intensity (e.g., 1 + , 2 + , 3 + ) is not provided in the laboratory report. Therefore, we were unable to retrospectively stratify results by band intensity.

The extended myositis panel at our centre tests for the following antibodies by line‐blot immunoassay: anti‐Mi2α, anti‐Mi2β, anti‐TIF1γ, anti‐MDA5, anti‐NXP2, anti‐SAE1, anti‐Ku, anti‐PM‐Scl100, anti‐PM‐Scl75, anti‐Jo‐1, anti‐SRP, anti‐PL‐7, anti‐PL‐12, anti‐EJ, anti‐OJ, and anti‐Ro52. For the purpose of this study, these were classified as either myositis‐specific autoantibodies (MSAs) or myositis‐associated autoantibodies (MAAs) according to established definitions, with MSAs including anti‐SRP, anti‐TIF1γ, anti‐Mi2α, anti‐Mi2β, anti‐MDA5, anti‐NXP2, anti‐SAE1, anti‐Jo‐1, anti‐PL‐7, anti‐PL‐12, anti‐EJ, and anti‐OJ, and MAAs including anti‐PM‐Scl75, anti‐PM‐Scl100, anti‐Ku, and anti‐Ro52.

Antinuclear antibodies (ANA) were detected using indirect immunofluorescence, with a positive result defined as a titre of ≥ 1:100. The extractable nuclear antigens (ENAs) panel included antibodies against Ro, La, Smith, U1RNP, Scl‐70, Jo‐1, and centromere proteins. These tests were conducted to assess for overlapping autoimmune conditions.

### 2.2 Variable Definitions

The diagnosis of ILD was based on high‐resolution computed tomography (HRCT) findings and clinical evaluation by a multidisciplinary team. ILD patterns were classified as usual interstitial pneumonia (UIP), nonspecific interstitial pneumonia (NSIP), organising pneumonia (OP), or diffuse alveolar haemorrhage (DAH) according to radiological criteria. Diagnosis of myositis was based on clinical assessment by a Rheumatologist, taking into account the clinical presentation, serum CK levels, imaging evidence of muscle inflammation on magnetic resonance imaging (MRI), electromyography (EMG), muscle histology, and response to treatment. The presence of malignancy was confirmed by histopathological diagnosis in the relevant cases.

Response to treatment was assessed retrospectively based on available clinical documentation, which included improvement in muscle strength, reduction in CK levels, improvement of extramuscular features (e.g., interstitial lung disease, rash), imaging or EMG findings (where available), and clinician judgment as documented in clinical notes.

## 3 Results

### 3.1 Demographics

Among the 25 patients with positive anti‐SRP antibodies, the mean age was 60.1 ± 16.1 years. The female‐to‐male ratio was 10:15. The ethnic distribution, based on electronic health records, was as follows: 17 White, six South Asian, one Black, and one South East Asian.

### 3.2 Clinical Features

The majority of patients (17/25) did not develop clinical features of myositis during their follow‐up period. In these patients, the duration from anti‐SRP antibody detection to data collection ranged from 0 to 57 months, with a median duration of 31 months. The primary reasons for requesting extended myositis panels in these patients included ILD, muscle complaints such as myalgia, Raynaud's phenomenon, or as part of investigations for known connective tissue diseases.

Out of the 25 positive cases, requests for anti‐SRP antibody testing were made by Rheumatology (14 cases), Respiratory (nine cases), Neurology (one case), and General Practice (one case). Of the eight patients subsequently diagnosed with myositis, six were requested by Rheumatology, one by Respiratory, and one by Neurology.

Of the eight patients diagnosed with myositis, the time from symptom onset to presentation varied between 3 weeks and 6 months. Six patients presented with proximal muscle weakness, while two presented with distal lower limb symptoms. None of the patients had a history of statin use or infection at disease onset. Table 1 shows the clinical features of the patients diagnosed with myositis.

**Table 1:** Clinical features of the eight patients with anti‐SRP antibody and myositis.

Patient	Duration from symptom onset to presentation		Pattern of weakness		Other relevant immunology	CK (IU/L)/MRI/EMG at presentation	Extramuscular manifestations	Histology (if available)	Malignancy	Therapy
	≤ 3 months	> 3 months	Proximal muscle	Distal muscle						
1	✓		✓		ANA 1:400 cytoplasmic/speckled, positive anti‐Ro52	CK 16377	Raised troponin, normal left ventricular function on echocardiogram, CT thorax showed minor inflammatory changes both lung bases	Myophagocytosis but no lymphocytic infiltrate, no increase in expression of membrane‐bound MHC Class I proteins	none	high dose steroids, then patient relocated so care transferred
2		✓	✓		ANA 1:1600 cytoplasmic/speckled, positive anti‐Ro52	CK 8643, MRI showed bilateral patchy proximal muscle oedema with muscle atrophy	Raynaud's phenomenon, early UIP pattern on CT thorax and restrictive pattern on lung function tests, normal echocardiogram	none	none	high dose steroids, MMF, currently on AZA and MTX
3		✓	✓		ANA 1:400 homogenous/speckled, positive anti‐TIF1 gamma	CK 493, MRI showed extensive symmetric muscle oedema in thighs, EMG findings in keeping with dermatomyositis	Dermatomyositis rash, echocardiogram showed moderately reduced left ventricular systolic function and extensive regional wall motion abnormalities	none	Two years later, diagnosed with T4 N1 M0 squamous cell lung cancer	high dose steroids, MTX and AZA
4		✓	✓		Anti‐Pm‐Scl75 positive, ANA negative	CK normal	none	none	none	MMF
5	✓			✓	anti‐SAE1 positive, ANA 1:400 homogenous	CK 617, MRI showed bilateral asymmetric myositis in lower legs, unilateral fascial oedema along Achilles tendon, EMG showed minor muscle irritability	CT thorax showed ILD‐NSIP pattern	No skeletal muscle abnormality on muscle biopsy	Five years later developed lung lesion suspicious for lung adenocarcinoma	MMF
6		✓		✓	ANA negative	CK normal, MRI showed muscle oedema in both calves. EMG after 3 months steroids was normal	none	No inflammation seen but biopsy limited due to fixation of the specimen	none	no response to MTX and AZA, so switched to MMF
7	✓		✓		ANA 1:400 homogenous/nucleolar, anti‐Ro52 positive	CK normal, MRI showed muscle oedema proximal muscles, EMG normal	CT thorax showed NSIP‐ILD, sclerodactyly, oesophageal dysmotility, Raynauds.	none	none	MMF, RTX, then ABT
8	✓		✓		ANA 1:400 homogenous, anti‐Jo‐1 and anti‐PM‐Scl100 positive	CK, EMG, and MRI upper arm normal	Sclerodactyly, Raynauds, mechanics hands, CT thorax showed CTD‐ILD	none	none	MMF, HQN

Abbreviations: ABT, abatacept; AZA, azathioprine; HQN, hydroxychloroquine; MMF, mycophenolate mofetil; MTX, methotrexate; RTX, rituximab.

#### 3.2.1 Interstitial Lung Disease

**Figure 1:**
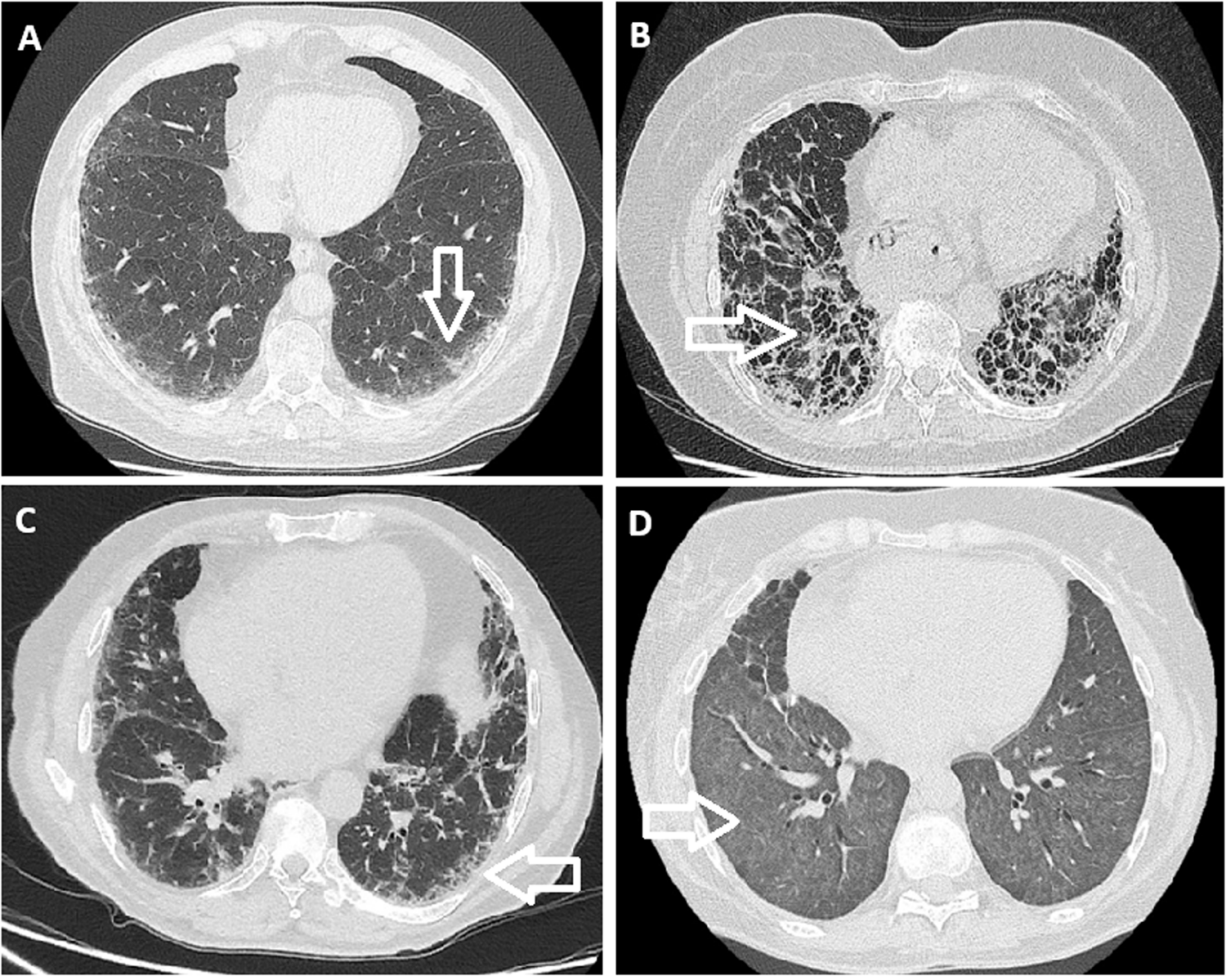
Example HRCT images of ILD in patients with anti‐SRP antibody. (A) Subpleural opacities in both lower lobes suggestive of NSIP pattern of ILD. (B) Bilateral peripheral reticulations, traction bronchiectasis, pleural‐parenchymal interface distortion and honeycombing, in keeping with UIP pattern of pulmonary fibrosis. (C) Fibrotic lung disease more in keeping with NSIP/OP than UIP. (D) Extensive bilateral ground‐glass opacification with interlobular septal thickening in keeping with pulmonary haemorrhage.

ILD was present 50% of patients with myositis and 53% of patients without myositis. Among the myositis patients with ILD, the identified patterns included UIP, NSIP, and OP. Figure 1 shows examples of these ILD patterns in selected patients. None of these patients had isolated anti‐SRP antibody positivity; they all exhibited additional autoantibodies, including Ro52, SAE‐1, PM‐Scl100, and Jo‐1.

Among the patients without myositis with ILD, the patterns identified were UIP, NSIP, DAH, and NSIP/OP overlap (see Figure 1). Two of these patients had isolated anti‐SRP antibody positivity, both with NSIP, while the remaining had additional antibodies, such as anti‐Ro52. One patient with DAH had lupus with multiorgan involvement.

## 4 Cardiac Involvement

Cardiac evaluations were available for four patients with positive anti‐SRP antibodies; two of whom had myositis. Among these:

- One patient had a raised troponin with a normal echocardiogram.
- The second patient exhibited moderately reduced left ventricular systolic function with extensive regional wall motion abnormalities and was also positive for TIF1‐gamma antibody.

## 5 Autoantibody Profile

We reviewed each patient's extended myositis panel to identify co‐existing myositis‐specific autoantibodies. Of the 25 patients, eight (32%) had an additional MSA, which included anti‐TIF1γ, anti‐SAE1, anti‐PL‐12, anti‐PL‐7, anti‐Mi2α, and anti‐Jo‐1. Twelve patients (48%) had an ANA titre ≥ 1:400, and eight were positive for anti‐Ro antibodies.

Among the eight patients diagnosed with myositis, three (38%) had an additional MSA, including anti‐TIF1γ, anti‐SAE1, and anti‐Jo‐1. Six patients (75%) in this subgroup had an ANA titre ≥ 1:400, and anti‐Ro positivity was observed in three patients.

Myositis‐associated autoantibodies (MAAs; excluding anti‐Ro) were also seen, with six patients (24%) demonstrating additional MAAs, including anti‐PM‐Scl75, anti‐U1RNP, anti‐PM‐Scl100, and anti‐Ku. Among those with myositis, two patients (25%) had an additional MAA: anti‐PM‐Scl75 and anti‐PM‐Scl100.

## 6 Creatine Kinase, Magnetic Resonance Imaging, and Electromyography Findings

CK levels were elevated in four of the eight myositis patients, with values of 493, 617, 8643, and 16,377 IU/L. The remaining four patients had normal CK levels. In two of these patients with normal CK, the diagnosis of myositis was supported by MRI findings of muscle oedema, while in the other two, the diagnosis was based on a compatible clinical picture and response to treatment.

**Figure 2:**
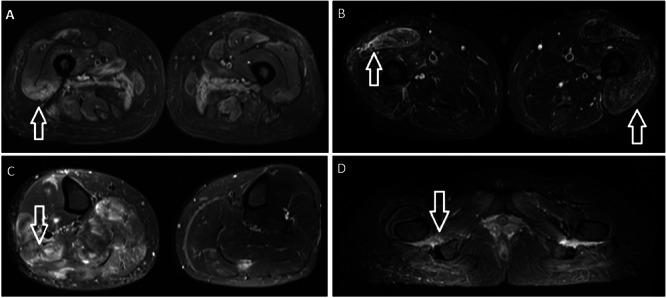
Example MRI scans showing myositis in patients with anti‐SRP antibody. (A) MRI thighs T2 image. Bilateral patchy areas of muscle oedema involving the abductor, adductor, extensor and hamstring muscles of the pelvis and both thighs. (B) Relatively symmetric oedema along the muscles in the anterior and posterior compartments of the thigh with relative sparing of the muscles in the posterior compartment. (C) Muscle oedema in both calves, more prominent on the right, without any atrophy/fatty infiltration. (D) Marked oedema in association with the quadratus femoris muscles.

MRI scans were performed in six of the eight myositis patients, revealing muscle oedema in five cases. The abnormalities were observed in the lower limbs (see Figure 2), with two patients showing oedema in the calves and three in the thighs. One patient exhibited both muscle oedema and atrophy.

EMG results were available for seven patients, five of whom were diagnosed with myositis. EMG findings were normal in three of these cases, including one patient who had already received steroid therapy. Of the two patients with abnormal EMG results, one demonstrated mild muscle irritability, and the other showed findings consistent with dermatomyositis. This latter patient was also positive for anti‐TIF1‐gamma antibody.

## 7 Histology Results

Muscle biopsy results were available for three patients with myositis. Findings included:

- One patient with CK 16,377 IU/L had biopsy results showing myophagocytosis without lymphocytic infiltrates or increased major histocompatibility complex class I expression.
- Another patient, with CK 617 IU/L and abnormal MRI and EMG findings, had a biopsy showing no skeletal muscle abnormalities. This patient was also positive for anti‐SAE1 antibody.
- The third patient's biopsy, though limited due to poor specimen fixation, showed no inflammation. This patient had isolated anti‐SRP antibody positivity and normal CK but demonstrated muscle oedema on MRI.

## 8 Presence of Malignancy

Two patients in the group of patients developed malignancy. One was a 66‐year‐old male ex‐smoker diagnosed with squamous cell lung cancer 2 years after myositis onset. This patient was also positive for anti‐TIF1‐gamma antibody. The second case involved a 64‐year‐old male smoker with a lung lesion suspicious for adenocarcinoma, identified 5 years after myositis onset. This patient was positive for anti‐SAE1 antibody.

## 9 Treatment

**Figure 3:**
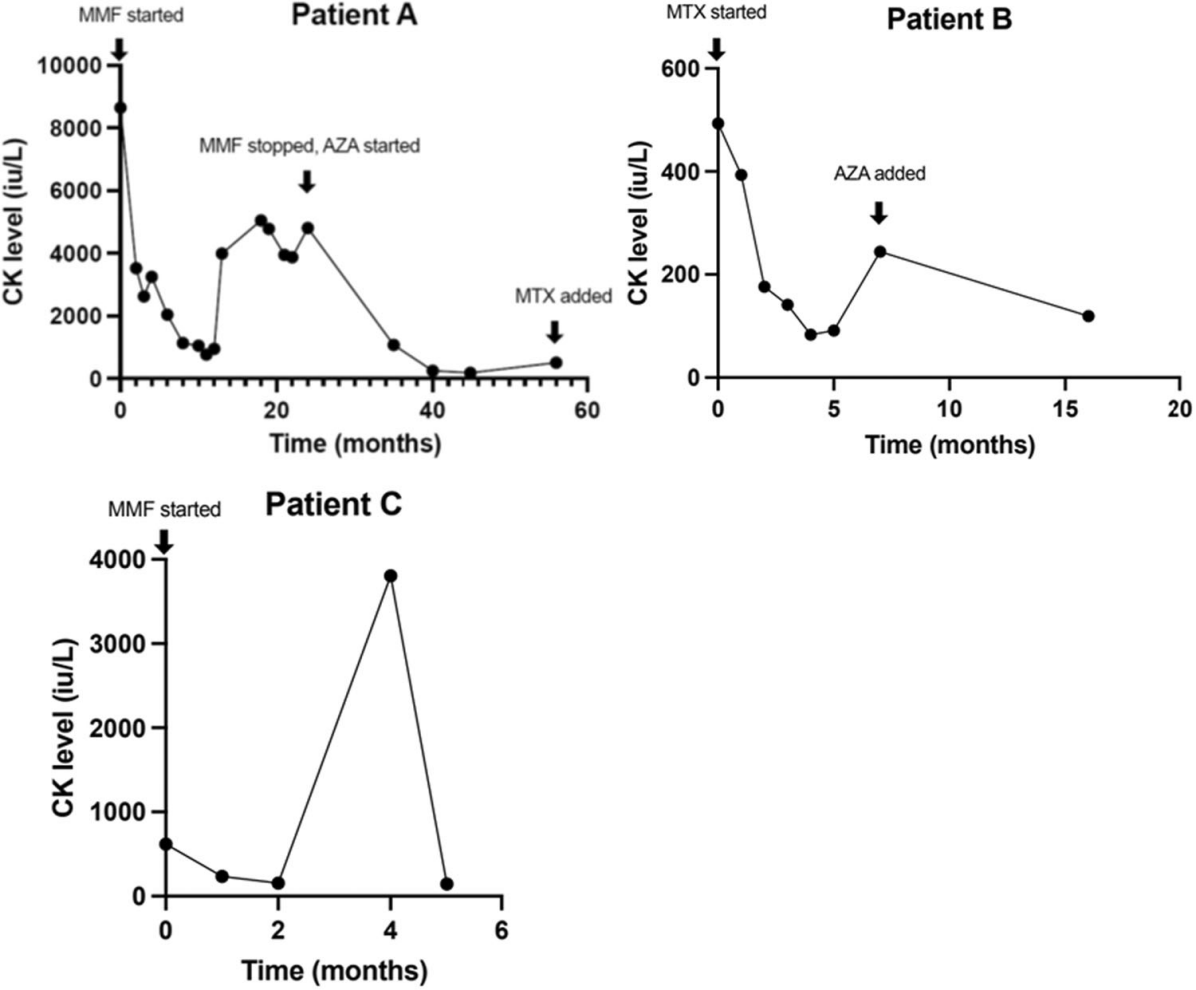
Response to treatment (based on CK level) for three patients with anti‐SRP myositis.

In the patients with myositis, the most commonly used disease modifying drug was MMF. Response to treatment for three patients is shown in Figure 3.

In those with normal CK at diagnosis, therapies used included MMF, HQN, AZA, RTX, and ABT. One patient had no response to the combination of MTX and AZA so was switched to MMF. Another patient had no response to MMF, so was switched to RTX and then ABT. In those patients with normal CK, treatment escalation was guided by muscle strength scores, and imaging. Other organ involvements were also considered when deciding on treatments, such as the presence of inflammatory arthritis, ILD, and cutaneous involvement.

## 10 Discussion

In this group of 25 patients with positive anti‐SRP antibodies, we observed a heterogeneous clinical presentation, with the majority (17/25) not developing clinical features of myositis during their follow‐up period. This finding is consistent with previous studies that highlight the potential for anti‐SRP antibody positivity without inflammatory myopathy [8], raising questions about the antibody's pathogenic role in certain cases. While anti‐SRP antibodies are primarily associated with IMNM, the variable clinical spectrum observed shows the need for careful patient monitoring and further investigation into factors that may trigger disease onset.

Among the eight patients who developed myositis, the clinical course was diverse, with differences in the pattern of muscle involvement, extramuscular manifestations, and response to treatment. Distal muscle symptoms were present in 2/8 of our patients, and this has also been reported in the literature [9]. Furthermore, patients with anti‐SRP antibody are more likely to have facial weakness [10], although this was not identified in any of our patients.

The presence of ILD in both myositis and non‐myositis patients was notable, with a total of 13 patients exhibiting ILD. This finding aligns with existing literature suggesting that anti‐SRP antibodies may be associated with ILD (Allenbach at al. [7]). The presence of overlapping autoantibodies complicates the interpretation of this association, although in our group of patients there was a subset of ILD patients without myositis who had isolated anti‐SRP antibody on the extended myositis panel. This entity has been reported [11], but there is a paucity of data in the literature on this subset of ILD. Suzuki et al.'s evaluation of 100 patients found ILD in 13%, although all of these patients had myositis [3]. One case series of three patients with ILD showed that immunosuppression was effective for these patients [12]. One case report described a patient with acute hypoxic respiratory failure secondary to anti‐SRP antibody‐associated interstitial lung disease [13]. Another case report described a patient with anti‐SRP antibody developing myositis after ILD [14]. Further studies are needed to delineate the specific contribution of anti‐SRP antibodies to ILD pathogenesis.

Cardiac involvement was seen in two of the eight myositis patients, although investigations were not uniformly carried out, and severity was variable between the cases. In the literature, cardiac involvement is also variable, with some studies showing a low risk of cardiac involvement [15], whereas one case described a patient developing cardiomyopathy and needing a heart transplant [16]. Given the potential for severe cardiac complications, routine cardiac assessment should be considered in these patients, particularly in those with symptoms suggestive of cardiac dysfunction.

We also found that anti‐SRP antibodies were associated with a high degree of overlap with other MSAs. While the coexistence of other MSAs with anti‐SRP antibody has been reported [17], one study looking at patients in the EuroMyositis registry found that only 0.2% of patients had more than one MSA (when tested using radiolabelled‐immunoprecipitation), demonstrating that MSAs are almost always mutually exclusive [18]. In our service evaluation, the presence of co‐existing autoantibodies does raise the possibility of antibody cross‐reactivity.

All patients were treated initially with steroids and then disease‐modifying drugs. Most patients in the group were treated with MMF. Other treatments included MTX, AZA, RTX, and ABT. There is a lack of randomised control trials to make formal recommendations on the treatment of IMNM. In the 224th ENMC International Workshop, treatments proposed include steroids, as well as MTX, and consideration of RTX and intravenous immunoglobulin [7]. Therapies must take into account patient comorbidities and organ involvement.

## 11 Limitations

Owing to the retrospective nature of this service evaluation, there was missing data for many patients. Due to the nature of clinical practice, there was a lack of homogeneity in the assessment and documentation of muscle strength, and investigations to determine the extent of extramuscular manifestations were not uniform for all patients, often being guided by patient symptoms and clinician preference. Investigations such as muscle biopsy were only performed in three patients. Overall, the small sample size limits the generalizability of the findings and precludes robust sub group analyses. Moreover, the high number of co‐existing autoantibodies in our group of patients made it difficult to ascertain whether certain clinical features were due to the pathogenic role of anti‐SRP or the other antibodies.

Additionally, the use of line‐blot immunoassays for antibody detection, while common in clinical practice, has known limitations in sensitivity and specificity. One study showed that anti‐SRP antibodies showed poor performance in predicting the IMNM‐like subtype using line blot [19].

Therefore, the possibility of false‐positive results cannot be excluded, particularly in patients without clinical manifestations. We were unable to determine antibody signal intensity (1 + , 2 + , 3 +) from the available laboratory records. Inclusion of weak positives, which may have lower specificity, could account for the high proportion of patients with co‐existing autoantibodies in our cohort, as well as the lower frequency of classic anti‐SRP IMNM features, including cardiac involvement.

Further studies are needed, utilising more specific methods such as immunoprecipitation, alongside advanced statistical techniques including regression modelling to adjust for potential confounders. This would help to enhance the robustness and generalisability of findings in this area.

## 12 Conclusion

This study highlights the heterogeneous clinical spectrum of anti‐SRP antibodies. While many patients with anti‐SRP positivity did not develop myositis, those who did exhibited diverse clinical courses and treatment responses. Correlating certain clinical features with specific autoantibodies is challenging when multiple MSAs are present. It also raises questions about the pathogenic role and clinical significance of anti‐SRP antibodies in certain cases, particularly when the results are provided by line‐blot immunosassay. Further research is needed to explain the mechanisms underlying anti‐SRP‐associated conditions and to optimise diagnostic and therapeutic strategies.

## Author Contributions

**Ameen Jubber:** conceptualization, investigation, writing – original draft, methodology, writing – review and editing, data curation, supervision, resources. **Maumer Durrani:** data curation, project administration, resources, visualization, writing – review and editing. **Abdullah Almayahi:** data curation, resources, project administration, visualization, writing – review and editing. **Kehinde Sunmboye:** data curation, resources, project administration, visualization, methodology, writing – review and editing.

## Ethics Statement

Ethics committee approval was received for this study under the Leicester, Leicesteshire and Rutland ethics committee of Institutional Review Board Number 14350.

## Consent

Consent was obtained from patients who participated in this study.

## Conflicts of Interest

The authors declare no conflicts of interest.
